# Effects of Linear-Polarized Near-Infrared Light Irradiation on Chronic Pain

**DOI:** 10.1100/2012/567496

**Published:** 2012-04-19

**Authors:** Dong Huang, Yong-Hong Gu, Qin Liao, Xue-Bin Yan, Shai-Hong Zhu, Chang-Qing Gao

**Affiliations:** ^1^Department of Anesthesiology, The Third Xiangya Hospital of Central South University, Changsha, Hunan 410013, China; ^2^Department of Pathology, The Third Xiangya Hospital of Central South University, Changsha, Hunan 410013, China; ^3^Research Center for Medical Sciences, The Third Xiangya Hospital of Central South University, TongZiPo Road 138, Changsha, Hunan 410013, China

## Abstract

In order to study the efficacy of linear-polarized near-infrared light irradiation (LPNIR) on relieving chronic pain in conjunction with nerve block (NB) or local block (LB), a 3-week prospective, randomized, double-blind, controlled study was conducted to evaluate the pre- and post-therapy pain intensity. Visual analogue scales (VASs) were measured in all patients before and 6 months after therapy visiting the pain clinic during the period of August 2007 to January 2008. A total of 52 patients with either shoulder periarthritis or myofascial pain syndrome or lateral epicondylitis were randomly assigned into two groups by drawing lots. Patients in Group I were treated with NB or LB plus LPNIR; Group II patients, for their part, were treated with the same procedures as in Group I, but not using LPNIR. In both groups, the pain intensity (VAS score) decreased significantly immediately after therapy as compared to therapy. There was a significant difference between the test and control groups immediately after therapy (*P* < 0.05), while no effect 6 months later. No side effects were observed. It is concluded that LPNIR is an effective and safe modality to treat various chronic pains, which has synergic effects with NB or LB.

## 1. Introduction

Nerve block (NB) or local block (LB) has conventionally been the main stream in treating chronic pain due to its reliable effects and convenience to apply. Repeated injections of the anesthetics and steroids at the same points, however, may cause local muscle damage and scarring. In addition, in many cases one-time injection may not provide a lasting effect to relieve chronic pain. Therefore, it is expected to develop a method to enhance the effects of NB or LB to obtain a better outcome, while reducing the times of injection. Recently, a noninvasive method, the linear-polarized near-infrared light irradiation (LPNIR), has been used effectively to relieve chronic pain from frozen shoulder, osteoarthritis, rheumatoid arthritis, postherpetic neuralgia, and other disorders [1–3]. We hypothesized that LPNIR in conjunction with NB or LB might provide better pain relief than NB or LB alone. Therefore, in the current study we evaluated the effect of LPNIR in conjunction with NB/LB in relieving chronic pain in the patients with shoulder periarthritis, back myofascial pain syndrome, or lateral epicondylitis.

## 2. Materials and Methods

A prospective, randomized, double-blinded study was conducted in our pain clinic from August 2007 to January 2008. Patients with chronic pain due to shoulder periarthritis, back myofascial pain syndrome, or lateral epicondylitis were randomly classified into two groups by drawing lots. Group I were treated with NB or LB plus LPNIR; Group II treated with the same procedures as in Group I, but without switching the machine on. A curtain was placed between the patient and the LPNIR machine, which is quite quiet when it is working. Therefore, both the patients and the administrator of the treatment did not know which arm the patient was in. The study protocol was approved by the Ethics Committee of the Third Hospital of XiangYa School of Medicine. An informed consent form was obtained from each patient after the subject was informed about the purpose of the study, the protocol, possible side effects of the administered agents, and risks and benefits of participation in the study.

Each potentially eligible subject who visited the pain clinic during the trial period was examined by the physician responsible for the trial to determine the suitability for inclusion in the study. Inclusion criteria were age > 18 years, chronic pain persisted for >3 months due to shoulder periarthritis, myofascial pain syndrome, or lateral epicondylitis, and with VAS scores > 5 (rating scale from 0 to 10). Patients with VAS scores < 5, or with existing contraindications for NB or LB, or simultaneously treated with other analgesic drugs or methods were excluded from participation in the study. Data from the patients that failed to follow up after the initiation of treatment from both of the study arms were analyzed using the last value before losing followup.

The following are the diagnostic criteria for the three diseases investigated in the current study:

**Shoulder Periarthritis:**

1. Patients around their fifties, more likely to be female than male, mostly physical laborers;
2. Pain around shoulders, especially at night and often chronic onset, always induced by weather changes and fatigue;
3. Movement of glenohumeral joint is limited by pain, which may gradually become less painful but more restricted in motion;
4. Atrophy of the shoulder muscle, tenderness in anterior, posterior and lateral aspects of the shoulder, marked limitation of shoulder abduction with typical shoulder carrying;
5. X-ray examination usually shows negative outcome, but osteoporosis may occur with chronic duration.

**Back Myofascial Pain Syndrome:**

1. Muscle pain with specific “trigger” or “tender” points which can be made worse with activity or stress. Trigger points can be identified by pressure to an area of a person's body;
2. Potential subjects can suffer from depression, fatigue, and behavioral disturbances.

**Lateral Epicondylitis:**

1. Pain that radiates from the outside of the elbow into the forearm and wrist;
2. Pain when touching or bumping the outside of the elbow;
3. A weak grip;
4. X-ray finding is negative.

## 3. Statistical Analysis

The differences between the two groups before therapy, and those within the same group obtained pre- and post-therapy, as well as the means of the differences pre- and post-therapy between the two groups—means of the treatment effects in the two groups were compared by unpaired or paired *t*-test using the program MedCalc version 11 (MedCalc Software Ltd., Mariakerke, Belgium) when it is applicable. The changes were estimated to be normally distributed. The effect of gender and age was also evaluated with the same method. A *P* value of <0.05 was considered significant.

## 4. Results

52 patients suffering from shoulder periarthritis, back myofascial pain syndrome, or lateral epicondylitis were enrolled in this study. Among them, there were 36 females and 16 males, age from 19 to 79 years old. By drawing lots, they were assigned randomly into two groups. The demographic data of the two groups were shown in Table 1.

In Group I, subjects received LPNIR near pain points 3 times a week as well as NB or LB once a week. The mixture of lidocaine (1%), bupivacaine (0.375%), and methylprednisolone acetate (0.25%) was used in LB or NB. In the cases of lateral epicondylitis and shoulder periarthritis, 4 mL and 6 mL of the mixture were injected into the pain points or “trigger” points, respectively, whereas in the back myofascial pain syndrome, the intercostal nerve involved was targeted with 8 mL of the mixture.

The LPNIR with a wavelength of 830 nm produced by LX-PZ99 (Zhuhai Concon Medical Instrument Co. Ltd., China) was set to the therapy model that irradiation circle was 2 : 3 (irradiation 2 seconds followed by 3-seconds pause). Each pain point was irradiated 10 min at 80% power output and treated for 3 weeks. The maximum power output is 1800 mW at a wavelength of 600 to 1600 nm.

In Group II (the control group), the medicines and the therapeutic procedures were the same as in Group I, just without switching on the LPNIR.

The pain relief effects were evaluated by comparing the visual analogue scales (VAS) scores prior to, immediately and 6 months after the treatment. VAS scores were graded as 0 with no pain and 10 as most pain imaginable.

The demographic data for the 52 subjects enrolled in the study were described in Table 1. There was no significant difference between these two groups.

The VAS scores of two groups at different time points were displayed in Table 2. There was no significant difference between the VAS scores of these two groups before therapy. In both groups, the average pain scores at posttherapy time point were significantly lower than that at pretherapy; there were significant differences between these two groups at posttherapy (Tables 2 and 3).

There was no significant difference in the effect at the point of 6 months after therapy that nearly reached the same levels at pretherapy (*P* > 0.05). There was no effect of age or gender on treatment effect. There was not enough data to analyze the effect of disease type. No thermal injury or other complications were observed in the study.

## 5. Discussion

The major finding of this study is that the LPNIR as an add-on method to NB or LB has synergistic effect on relieving pain of shoulder periarthritis, myofascial pain syndrome, or lateral epicondylitis in the acute phase.

Previous data show that LPNIR is safe and effective in relieving chronic pain and other conditions. Basford et al. [4] investigated the safety and effects of LPNIR in a two-phase study, in which they first established its safety in 6 adults with normal neurological examination by transcutaneous irradiation with LPNIR; then they demonstrated in a double-blind study consisted of 12 subjects with upper extremity complex regional pain syndrome I, showing that irradiation can induce more than 50 percent pain reduction. Demura et al. [2] found that LPNIR can significantly increase the ranges of the shoulders and ankle motion in the treated group when compared with the placebo. Yokoyama and Oku [5] first treated 4 patients with rheumatoid arthritis-affected temporomandibular joint pain and who were refractory to antirheumatic agents, obtained satisfied pain relief with only four treatments. They later observed the same effects in 20 patients who were suffering from temporomandibular pain due to nonrheumatic arthritis and who were without attenuation after conservative treatments [1]. Mibu et al. [6] treated 35 patients and found that LPNIR is a safe and effective measure for intractable anorectal pain.

In line with these findings, we found that LPNIR had synergic effect with NB or LB in treating chronic pain from shoulder periarthritis, back myofascial pain syndrome, or lateral epicondylitis.

This is within expectation in view of the following findings: first, LPNIR treatment near the stellate ganglion results in the increase of blood flow velocity similar to that caused by stellate ganglion block [7]; second, double-blinded study showed that LPNIR induces warmth in the treated areas [4].

Local heating has long been proved to have pain-relieving effects and can increase elasticity of connective tissue and joint range [8, 9]. The mechanisms involved in it may include increasing local blood flow and enhancing mild inflammatory reaction mediated through the release of histamine and prostaglandins; promoting vasodilatation; changing enzyme activity and metabolic rate; increasing pain threshold through a direct action of heat upon free nerve endings or nerve trunk that supply the affected area by various mechanisms, including impacting on neuronal membrane potential [8, 10]. Laser irradiation, which also causes warm sensation, has indeed been proved to decrease sensory nerve conduction velocity and compromise the response of dorsal horn neurons to formalin [11, 12].

The infrared light is a form of electromagnetic radiation with wavelengths longer than visible light, which is generated by objects that produce heat, such as human beings. According to its wavelength, infrared light is subdivided into: near-, mid-, and far-infrared light. Polarization can give it a predictable fashion with respect to the propagation direction in which way energy is focused.

The infrared rays with wavelength of 600–1600 nm in LPNIR are capable of reaching the deeper tissues, not readily reflected, dispersed, or absorbed at the surface of the body [13], and the energy light is partially converted to thermal energy and produces warming sensation.

Indeed, Kashima et al. [3] observed changes in sensory and pain perception thresholds in the trigeminal region, and Muneshige et al. [14] observed antinociceptive effects in vivo in rats after the treatment of LPNIR.

It seems that in our settings the effects of the treatment can last no longer than 6 months.

In accordance with the previous observations, we did not observe any side effects related to the treatment of LPNIR in any of our patients.

The limitation of this study is that the number of patients of each type of diseases is too small to allow us to draw a conclusion on the effect of disease type. Further larger trial is needed to clarify this.

## 6. Conclusions

Our study shows that LPNIR is an effective and safe modality to treat various chronic pains, which has synergic effects with NB or LB.

## Figures and Tables

**Table 1:** Demographics of enrolled subjects in the two groups.

	Group I (block + LPNIR)	Group II (block only)
Gender:		
Male	7 (26.9%)	9 (34.6%)
Female	19 (73.1%)	17 (65.4%)
Age (mean ± SD)	53.69 ± 13.01	45.73 ± 13.49
Type of chronic pain:		
Shoulder periarthritis	10 (38.5%)	8 (30.8%)
Myofascial pain syndrome	13 (50.0%)	12 (46.2%)
Lateral epicondylitis	3 (11.5%)	6 (23.1%)
Total cases	26	26

SD: standard deviation.

**Table 2:** The VAS scores pre- and immediately post-therapy in two groups.

	Pretherapy (mean ± SE)	Posttherapy (mean ± SE)
Group I	7.77 ± 0.28	1.54 ± 0.31^#^
Group II	8.00 ± 0.28^†^	3.08 ± 0.41^#^

^†^ *P* > 0.05 as compared with Group I; ^#^ *P* < 0.005 as compared with pretherapy.

SE: standard error.

**Table 3:** Means of differences between pre- and immediately post-therapy.

Group	Differences (treatment effects) (mean ± SE)
I	6.23 ± 0.45
II	4.92 ± 0.42*

*P < 0.05 as compared with Group I; SE: standard error.

## References

[1] Yokoyama K, Sugiyama K (2001). Temporomandibular joint pain analgesia by linearly polarized near-infrared irradiation. *Clinical Journal of Pain*.

[2] Demura S, Yamaji S, Ikemoto Y (2002). Effect of linear polarized near-infrared light irradiation on flexibility of shoulder and ankle joints. *Journal of Sports Medicine and Physical Fitness*.

[3] Kashima K, Watanabe N, Higashinaka S, Maeda S, Shiba R (2005). Changes in sensory and pain perception thresholds after linear polarized near-infrared light radiation in the trigeminal region. *Cranio*.

[4] Basford JR, Sandroni P, Low PA, Hines SM, Gehrking JA, Gehrking TL (2003). Effects of linearly polarized 0.6-1.6 *μ*M irradiation on stellate ganglion function in normal subjects and people with complex regional pain (CRPS I). *Lasers in Surgery and Medicine*.

[5] Yokoyama K, Oku T (1999). Rheumatoid arthritis-affected temporomandibular joint pain analgesia by linear polarized near infrared irradiation. *Canadian Journal of Anaesthesia*.

[6] Mibu R, Hotokezaka M, Mihara S, Tanaka M (2003). Results of linearly polarized near-infrared irradiation therapy in patients with intractable anorectal pain. *Diseases of the Colon and Rectum*.

[7] Lee SC, Lim YJ, Han SM The change of blood flow velocity of radial artery after linear polarized infrared light irradiation near the stellate ganglion comparing with stellate ganglion block.

[8] Goats GC (1989). Continuous short-wave (radio-frequency) diathermy. *British Journal of Sports Medicine*.

[9] Nuhr M, Hoerauf K, Bertalanffy A (2004). Active warming during emergency transport relieves acute low back pain. *Spine*.

[10] Carpenter DO (1967). Temperature effects on pacemaker generation, membrane potential, and critical firing threshold in Aplysia neurons. *Journal of General Physiology*.

[11] Snyder-Mackler L, Bork CE (1988). Effect of helium-neon laser irradiation on peripheral sensory nerve latency. *Physical Therapy*.

[12] Shimoyama M, Fukuda Y, Shimoyama N, Iijima K, Mizuguchi T (1992). Effect of He-Ne laser irradiation on synaptic transmission of the superior cervical sympathetic ganglion in the rat. *Journal of Clinical Laser Medicine and Surgery*.

[13] Mezitis M, Rallis G, Zachariades N (1989). The normal range of mouth opening. *Journal of Oral and Maxillofacial Surgery*.

[14] Muneshige H, Toda K, Ma D, Kimura H, Asou T, Ikuta Y (2006). Antinociceptive effect of linear polarized 0.6 to 1.6 *μ*m irradiation of lumbar sympathetic ganglia in chronic constriction injury rats. *Journal of Rehabilitation Research and Development*.

